# Proteomic Analysis of Nasal Epithelial Cells from Cystic Fibrosis Patients

**DOI:** 10.1371/journal.pone.0108671

**Published:** 2014-09-30

**Authors:** Ludovic Jeanson, Ida Chiara Guerrera, Jean-François Papon, Cerina Chhuon, Patricia Zadigue, Virginie Prulière-Escabasse, Serge Amselem, Estelle Escudier, André Coste, Aleksander Edelman

**Affiliations:** 1 Service de Génétique et Embryologie Médicales, Unité Mixte de Recherche_Scientifique 933, Institut National de la Santé et de la Recherche Médicale, Université Pierre et Marie Curie – Paris 6, and Assistance Publique – Hôpitaux de Paris, Hôpital Armand Trousseau, Paris, France; 2 Unité_1151, Institut National de la Santé et de la Recherche Médicale, Université Paris Descartes, Paris, France; 3 Plateau Proteome Necker, Structure Fédérative de Recherche de Necker, Université Paris Descartes, Paris, France; 4 Unité Mixte de Recherche_Scientifique 855, Institut National de la Santé et de la Recherche Médicale, Université Paris 12, Faculté de Médecine, Créteil, France; 5 Service d’Otorhinolaryngologie et de chirurgie cervico-faciale, Assistance Publique – Hôpitaux de Paris, Hôpital inter-communal et Groupe Hospitalier Henri Mondor-Albert Chenevier, Créteil, France; Shantou University Medical College, China

## Abstract

The pathophysiology of cystic fibrosis (CF) lung disease remains incompletely understood. New explanations for the pathogenesis of CF lung disease may be discovered by studying the patterns of protein expression in cultured human nasal epithelial cells (HNEC). To that aim, we compared the level of protein expressions in primary cultures of HNEC from nasal polyps secondary to CF (CFNP, n = 4), primary nasal polyps (NP, n = 8) and control mucosa (CTRL, n = 4) using isobaric tag for relative and absolute quantification (iTRAQ) labeling coupled with liquid chromatography (LC)-MS-MS. The analysis of the data revealed 42 deregulated protein expressions in CFNP compared to NP and CTRL, suggesting that these alterations are related to CF. Overall, AmiGo analysis highlighted six major pathways important for cell functions that seem to be impaired: metabolism, G protein process, inflammation and oxidative stress response, protein folding, proteolysis and structural proteins. Among them, glucose and fatty acid metabolic pathways could be impaired in CF with nine deregulated proteins. Our proteomic study provides a reproducible set of differentially expressed proteins in airway epithelial cells from CF patients and reveals many novel deregulated proteins that could lead to further studies aiming to clarify the involvement of such proteins in CF pathophysiology.

## Introduction

Chronic inflammation, which represents one of the most serious causes of morbidity/mortality in the world, is a severe complication common to both frequent and rare disorders. Among chronic inflammatory disorders, cystic fibrosis (CF) is the most common recessively inherited disease in the Western world. CF is caused by mutations in the gene encoding the cystic fibrosis transmembrane conductance regulator (CFTR) protein [1], which is expressed in airway epithelial cells and acts as a chloride channel activated by cyclic AMP. The most frequent mutation of CF alleles is F508del, a deletion of phenylalanine in the intra-cytosolic nucleotide binding domain 1 of CFTR.

Primary nasal polyposis, a common disorder of the upper airways [2] which affects almost 4% of the population, is an inflammatory disease of unknown etiology. Interestingly, CF is often associated with nasal polyps [3] that constitute an accessible model to study CF pathophysiology.

Global approaches may be useful to unmask abnormal cellular pathways in complex chronic inflammatory diseases. A few proteomic studies have been performed in CF with different sample sources: sputum [4], [5], bronchio-alveolar lavage fluid [6] or serum [7]. Only three global proteomic studies have been performed in airway epithelial cells which are one of the main targets in CF: two in lung epithelial cell lines IB3-1 (i.e. immortalized bronchial epithelial cell line isolated from a patient homozygous for the F508del mutation) and C38 (i.e. IB3-1 “corrected” by transfection with wild type *CFTR*) [8], [9] and the last one in cells obtained by nasal brushing (including blood, inflammatory and epithelial cells) from CF patients [10]. To our knowledge, no proteomic studies have been done on primary cultures of pure airway epithelial cells from CF patients (i.e. not contaminated with blood and/or inflammatory cells). This culture system allows epithelial cell differentiation at the air/liquid interface and is useful to study chronic inflammation in airways [11].

The effects of F508del CFTR are complex at the cellular level and are known to alter many different functions [11]–[13]. Among them, metabolism, G protein process, inflammation, oxidative stress response, protein folding, proteolysis and structural proteins are known to be abnormal but the proteins implicated in those malfunctions are largely unknown.

The aim of the present study was to compare the levels of protein expressions in primary cultures of human nasal epithelial cells (HNEC) from control mucosa (CTRL), primary nasal polyps (NP) and NP secondary to CF (CFNP). We chose differential quantitative global proteomic analysis based on isobaric tag for relative and absolute quantification (iTRAQ) labeling coupled with liquid chromatography (LC)-MS-MS that permitted reproducible protein quantification in the different patients evaluated in this study. The iTRAQ method is based on the covalent labeling of the amines from proteins with isobaric tags containing a different distribution of heavy isotopes. The fragmentation of the attached tag generates a low molecular mass reporter ion that can be used to relatively quantify the peptides and the proteins from which they originated.

The results provided new insights into the comprehension of abnormal airway epithelial cell functions that might exist in CF pathogenesis.

## Materials and Methods

### Subjects

**Figure 1:**
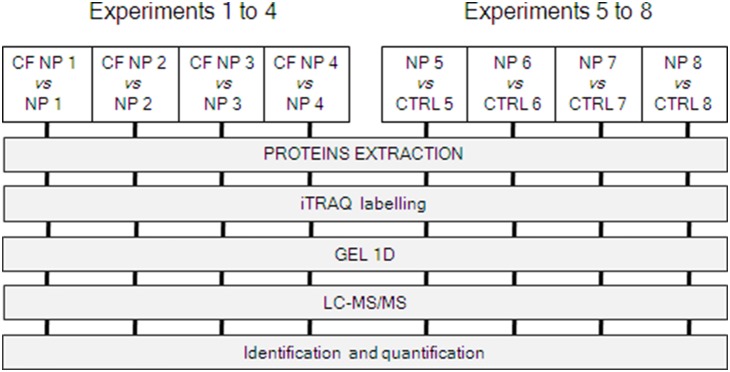
Workflow for the study. Two series of independent experiments were compared: (i) experiments 1 to 4 for proteins derived from HNEC cultures obtained from 4 CF NP patients *vs.* 4 NP patients (non CF); (ii) experiments 5 to 8 for proteins derived from HNEC cultures obtained from 4 NP patients (non CF) *vs.* 4 controls patients. Each couple of samples was matched randomly. For quantification of proteins, iTRAQ labeling coupled to LC-MS/MS was performed (see methods for details).

In order to evaluate the differential protein expression related to CF four patients with CFNP were randomly matched to four non-CF patients affected by NP (CFNP *vs.* NP), and four other non-CF patients with NP were matched to four CTRL patients without nasal polyposis (NP *vs.* CTRL) (Figure 1).

All NP samples were collected from patients requiring surgery for their nasal polyposis. Patients were requested to stop all treatments at least one month before surgery except for CF patients who were prepared for surgery by systemic antibiotics adapted to nasal bacteriology. Four control mucosa from the inferior turbinate were obtained from individuals who underwent turbinectomy for snoring. All the patients did not mention any allergic background and were negatively tested with skin prick tests. All non-CF patients with NP presented typical primary nasal polyposis and did not suffer from recurrent airway infections. In order to avoid, as much as possible, heterozygous carriers of the F508del mutation, nasal polyps occurring in a context of purulent chronic sinusitis were not included [14]. All four CF patients were homozygous for the F508del mutation. The CTRL and NP patients were not genotyped for CF mutations. Characteristics of the patients are given in Table 1. This protocol was approved by the ethics committee of our institution (Comité de Protection des Personnes-Ile-de-France IX CHU Henri Mondor). The participants provided their verbal consent to participate in the study and their non opposistion to the use of their data. Parental consent was obtained for minor subjects. The nasal mucosa collected for the experiments were either nasal polyps or turbinates obtained during ethmoidectomy or turbinectomy, respectively. All these samples were surgical waste, usually discarded during surgery. Our procedure is based on French law n°2004-800 of 6 August 2004 on Bioethics (“loi relative à la bioéthique”) which was codified in the French Public Health Code (L. 1235-2 and L. 1245-2). The surgeon recorded in the patient’s file the non-opposition for using the discarded nasal mucosa.

**Table 1:** Description of patients.

Patient	Sex	Age (years)	Associated disease	Cigarette smoke
**CTRL**				
CTRL 5	M	32	None	No
CTRL 6	F	24	None	No
CTRL 7	M	28	None	No
CTRL 8	M	20	None	Yes
**CFNP** *				
CFNP 1	M	21	None	No
CFNP 2	F	15	None	No
CFNP 3	M	22	None	No
CFNP 4	F	25	None	No
CFNP 5	F	19	None	No
CFNP 6	M	21	None	No
CFNP 7	M	17	None	No
**NP**				
NP 1	M	25	Samster’s disease	No
NP 2	F	29	Aspirin intolerance	Yes
NP 3	M	35	Asthma	No
NP 4	F	34	Asthma	No
NP 5	M	33	Samster’s disease	No
NP 6	F	23	Asthma	No
NP 7	M	37	Asthma	No
NP 8	F	41	Samster’s disease	No

*All CFNP patients are homozygous for the F508del *CFTR* mutation.

### Material

#### Reagents for mass spectrometry and protein biochemistry experiments

dithiothreitol (DTT), pronase, ethylenediamine tetra-acetic acid (EDTA), collagen IV, urea, thio-urea, orthovanadate, glycerol, TRIS, iodoacetamide, NaCl, detergents (CHAPS, sodium deoxychotate, Triton X100), NH4CO3 and the Glu-fibrinopeptide were from Sigma-Aldrich (Saint-Quentin Fallavier, France). Anti-proteases were from Roche (Neuilly sur Seine, France). Bromophenol blue, SDS, acrylamide, silver staining kit were from Biorad (Marnes la Coquette, France). Trypsin for mass spectrometry was of highest grade purchased from Promega (Charbonnieres-les-bains, France). Acetonitril (ACN) and Tri-fluoro-acetic acid (TFA) were from Carlo Erba (Val de Reuil, France), Alpha-Cyano-4-Hydroxycinnamic Acid (HCCA) from Laser Biolabs (Sophia-Antipolis, France). iTRAQ reagents were from Applied Biosystems (Courtaboeuf, France)

#### Columns

C18 trap column (C18 pepmap100, 5 µm particles, 100 Å pores, 300 µm i.d., 5 mm length), and C18 analytical column (C18 pepmap100, 3 µm particles, 15 cm length, 75 µm inner diameter., 100 Å pores) were from Acclaim (Vandoeuvre, France). The Ultimate3000 series HPLC and the Probot fraction collector were purchased from Dionex (Voisins le Bretonneux, France).

### Primary cultures of HNEC

Nasal samples were transported to the laboratory in order to perform primary cultures of HNEC as previously described [11]. Mucosa samples were rinsed in phosphate-buffered saline (PBS) with dithiothreitol (DTT, 5 mM) and antibiotics (100 U/ml of penicillin, 100 mg/ml of streptomycin, 2.5 µg/ml of amphotericin B and 100 mg/ml of gentamicin) and then placed overnight at 4°C in a PBS-antibiotics solution containing 0.1% pronase. The samples were incubated in DMEM/F12 with 5% fetal calf serum (FCS) before centrifugation (800 g, 7 min). Cell pellets were then suspended in 0.25% trypsin-EDTA solution for 3 min and incubated in DMEM/F12-antibiotics with 10% FCS. Finally, HNEC were plated on permeable polycarbonate supports at 1×10^6 ^cells/cm^2^. All inserts had a diameter of 12-mm and were coated with type IV collagen. HNEC were incubated at 37°C in 5% CO_2_. For the first 24 h, HNEC were incubated with 1 ml of DMEM/F12-antibiotics with 2% Ultroser G outside the insert and DMEM/F12-antibiotics with 10% FCS inside the insert. After 24 h, medium was removed inside the inserts in order to place the cells at an air-liquid interface, and medium outside the inserts was then changed daily. Transepithelial resistance and potential difference were measured every three days using a microvoltmeter (World Precision Instruments, Astonbury, UK). The primary culture of HNEC, differentiated at the air-liquid interface, provides functional cells closely resembling normal airway epithelium within one week that maintain their differentiation for at least four weeks [15]. We previously controlled that all cultured cells are epithelial cells, with viability around 95% during the first four weeks. For all experiments the cells were studied at day 7 after seeding, corresponding to the establishment of cell differentiation.

### Protein preparation for proteomic experiments

Experiments for protein quantification were performed in primary cultures of HNEC from CFNP, NP and CTRL. Cells in Transwell filters were washed, scraped off the filters in ice-cold PBS, and centrifuged (800 g, 10 min, 4°C). The pellet was suspended in 100 µl/culture plate of lysis buffer, constituted of 0.5% w/v SDS, 50 mM Tris-HCl pH 7, before incubation at 95°C for 5 min. The total cellular proteins obtained were supplemented by 250 µl/culture plate of dissolution buffer, constituted of 8 M urea, 2.5 M thio-urea, 4% w/v CHAPS, 50 mM DTT, anti-protease 1X, 1 mM orthovanadate, and mixed one h at room temperature. Labeling of proteins with iTRAQ reagents, protein fractionation and digestion were performed according to previously described protocols [16].

### iTRAQ labeling

After protein preparation, 25 µg of each sample were precipitated overnight at −20°C using 100% acetone and dissolved in 20 µl of denaturing buffer containing 1 µl denaturant and 2 µl reducing reagent (TCEP) as provided in the iTRAQ kit, followed by vortexing and incubation at 60°C for 1 h. One µl of cysteine blocking solution (iodoacetamide) was then added to each sample, followed by incubation at room temperature for 30 min. iTRAQ labeling was carried out by adding iTRAQ reagents 114, 115, 116 or 117 to either the NP, CF NP or CTRL samples followed by incubation at room temperature for 2 h. For the NP/CFNP experiments, iTRAQ reagent 114 was used for NP and the 116 was used for CFNP. For the NP/CTRL experiments: iTRAQ reagent 115 was used for NP and the 117 was used for CTRL. Subsequently, two samples/experiment (NP/CF NP or NP/CTRL) were mixed by vortexing, pooled together and dried in a Speedvac evaporator (Thermo Fisher Scientific, Courtaboeuf, France).

### Trypsin digestion

Samples were digested according to Bensalem et al [17] with minor modifications. Briefly, prior to mass spectrometry analysis, the dry iTRAQ sample pellet was dissolved in Laemmli buffer, constituted of 10% glycerol, 0.05 M Tris-HCl (pH 6.8), 0.1% SDS, 50 mM DTT, and 0.01% (w/v) bromophenol blue, then supplemented by 1/5 H_2_O dilution. Dissolved proteins were separated by electrophoresis (SDS-PAGE). Briefly, protein samples were loaded onto 10% w/v polyacrylamide gels and run at 25 mA for 2 to 3 h at room temperature. After electrophoresis, protein visualization was carried out by silver staining compatible with MS/MS analysis.

12 protein bands were excised from the SDS-PAGE gel and prepared as follows: each gel piece was destained for 5 min with 200 µl of destaining buffer (silver staining kit). Before drying the gel pieces in a Speedvac evaporator, they were washed with de-ionised water (Millipore, Guyancourt, France) and shrunk in the presence of Acetonitril for 15 min. The gel pieces were dried at room temperature for 20 min and followed by digestion with 0.8 µg (4 U) of trypsin in 8 µl of 25 mM NH_4_/CO_3_, overnight at 37°C. The peptide mixture was extracted from the gel plug with 10 µl of a 50% ACN, 0.1% tri-fluoro-acetic acid solution and dried in a Speedvac evaporator.

### Nano-LC-MALDI-TOF-TOF

#### Nanochromatography protocol

Digested peptides were suspended in water with 0.1% TFA and 10% acetonitrile, separated with an Ultimate3000 series HPLC. Briefly: 10 µL of eluate were injected and trapped using solvent A (0.1% Trifuoroacetic acid, 2% ACN) at a 30 µl/min loading flow for 3 min in a C18 trap column. The microvalve then oriented the elution to back flow (300 nl/min) the peptides toward the analytical column with a gradient rising from 7% solvent B (80% acetonitrile, 20% solvent A) at microvalve switch to 16% in 7 min, from 16% to 32% in 20 min and from 32% to 50% in 7 min. Fractions were spotted on-line on a MALDI target using a Probot fraction collector. Spotted fractions were mixed 1∶4 with 2 mg/ml of HCCA in 70% ACN 0.1% TFA and Glu fibrinopeptide at 30 fmoles/spot. 96 fractions were collected and analysed using a 4800 MALDI TOF/TOF analyser (ABI, Lyon - Rhône-Alpes, France).

#### MS mode

Spectra acquisition and processing were performed using the 4000 series explorer software (ABI) version 3.5.28193 build 1011 in positive reflectron mode at fixed LASER fluency with low mass gate and delayed extraction. External plate calibration was performed using 4 calibration points spotted throughout the plate, additional internal calibration was performed using the Glu-fibrinopeptide (M/Z = 1570.677) allowing a mass accuracy of few ppm. For each fraction, steps of 50 spectra in the range of 700 to 4000 Da were acquired at a 200 Hz LASER shot frequency. 500 spectra per sample were summed and processed to obtain monoisotopic values from isotopes clusters with a raw spectra s/n ratio of 20.

#### MS/MS mode

In each MS spectrum, the 8 most abundant peaks were selected for fragmentation starting with the least abundant. A cut off was applied at a minimum s/n of 20. Neighboring precursors within resolution of 200 were excluded. 1000 MS/MS spectra per precursor were summed by increments of 50. Processing included baseline subtraction and Stavitsky Golay smoothing with 15 points across peak and a polynomial order of 4. Peaklists reflect monoisotopic values from isotope clusters with a s/n ratio of minimum 22.

#### Peak list and quantification analysis

Generated MS/MS peaklists were subsequently submitted to an in-house Mascot version 2.2 search engine (Matrix science). The Swiss-Prot 55.5 release Swiss-Prot 20080610 (389046 sequences; 139778124 residues) release database was used with the Homo sapiens species. Parent and fragment mass tolerances were respectively set to 50 ppm and 0.3 Da. Variable modification, oxidation (M), iTRAQ 4plex (Y) and fixed modification, iTRAQ 4plex (N-term), iTRAQ 4plex (K), carbamidomethylation (C) were allowed. Trypsin digestion and possibility of one missed cleavage were selected. A filter was applied to the search in order to reduce false positives and matching redundancies of the same peptide in several hits. All matches above 1% risks of random matching were eliminated (p<0.01). The probability score calculated by the software was used as a primary criterion for correct identification. For Mascot scores slightly above the threshold of p value, measurement errors were carefully watched to detect possible false positives that usually give random mass measurement errors, while relevant identifications give constant measurement errors. Molecular weights from experimental and Mascot results were also compared. Whenever the result was ambiguous, the spectra either in MS or MS/MS were manually checked for proper matching and the result was accordingly rejected or accepted.

### Statistical analysis for proteomic experiments

For the quantification, the protein ratio type was the ‘weighted’ geometric mean, only ‘bold face ratios’ were considered (significantly different from 1 at a 95% confidence level), outlier removal was ‘automatic’ (Dixon’s method and Rosner’s method), the minimum number of peptides was two and at least two peptides were required to be the top ranking peptide matches (‘bold and red type faces’). Selective criteria were adopted to obtain a consistently quantified set of proteins: (i) the intensity ratio must be calculated with at least two unique quantified peptides and (ii) the protein must be quantified in at least 3/4 experiments. Differences between expression of proteins in CFNP vs NP were evaluated by Mann-Whitney test p-value<0.05 (mostly confirmed by Student test with Aspin-Welch correction, p-value<0.05) (Table S1). Proteins were therefore considered up/down-regulated if the average of the ratio of the protein expression were >1.37 and <0.73. To determine this threshold, we calculated the standard deviations of the protein ratios for each experiment, we normalized the Gaussian distributions and we calculated the average threshold to select the proteins upregulated (last 20% on the right of the Gaussian) and the more downregulated under-expressed (first 20% on the left of the Gaussian). These thresholds are in agreement with other studies using iTRAQ labeling [18]. Finally, the reliably quantified protein data set for both series of experiments were analysed using the AmiGO toolbox kit (http://amigo.geneontology.org/cgi-bin/amigo/term_enrichment and Cytoscape software).

### SDS PAGE and Western blot

All Western blot analyses were performed according to previously described protocols [11]. Analysis of protein expression in HNEC was performed using antibodies directed against human STIP1, TERA, EZRI, ANXA1, 1433S, KPYM and HSPB1. All the used primary and secondary antibodies are listed in the supplemental data with all the details including stock concentrations, used dilutions, manufacturer details and specificity (Table S2). For Western blots realized from NP 1, 2, 3, 4 and CFNP 3, 5, 6, 7, experiments were performed on four patients per category and in triplicate. Mann and Whitney test was used for comparison of data series and P values≤0.01 were considered statistically significant. Data are expressed with Box plots including median, maximum and minimum values and first and third quartiles.

#### SDS PAGE

Proteins were prepared as follows: first, HNEC from CF NP, NP and CTRL in Transwell filters were washed twice, scraped off the filters in ice-cold PBS, and centrifuged at 800 g for 10 min at 4°C. Second, the pellet was re-suspended in 500 µl of RIPA lysis buffer constituted of 150 mM NaCl, 50 mM Tris-HCl (pH 7.6), 1% v/v Triton X-100, 0.1% w/v SDS, 1% w/v sodium deoxychotate and antiprotease 1X, and kept on ice for 30 min under agitation. Third, the cell lysates were then centrifuged (20 000 g, 15 min) at 4°C. Finally, samples of the supernatants (20 µg protein in 1 volume Laemmli buffer containing 10% glycerol, 0.05 M Tris-HCl (pH 6.8), 0.1% SDS, 50 mM DTT, and 0.01% (w/v) bromophenol blue) were loaded onto 10% w/v polyacrylamide gels and run at 25 mA for 2 to 3 h at room temperature and electrically transferred at 200 mA for 90 min to nitrocellulose membranes.

#### Western blot analysis

The proteins of interest were detected using appropriate first and secondary antibodies all used at 1∶15000 dilution. The infra-red signal was obtained by scanning with the Odyssey LI-COR (Techsciences, Cergy Saint Christophe, France). Quantification of protein levels was obtained using the Odyssey LI-COR software. For normalization, the acrylamide gels were stained with Coomassie blue (total migrated protein detection) just after electro-transfer [19] and scanned using the Odyssey LI-COR.

## Results

### Comparison of protein expression patterns in CFNP, NP and CTRL

**Figure 2:**
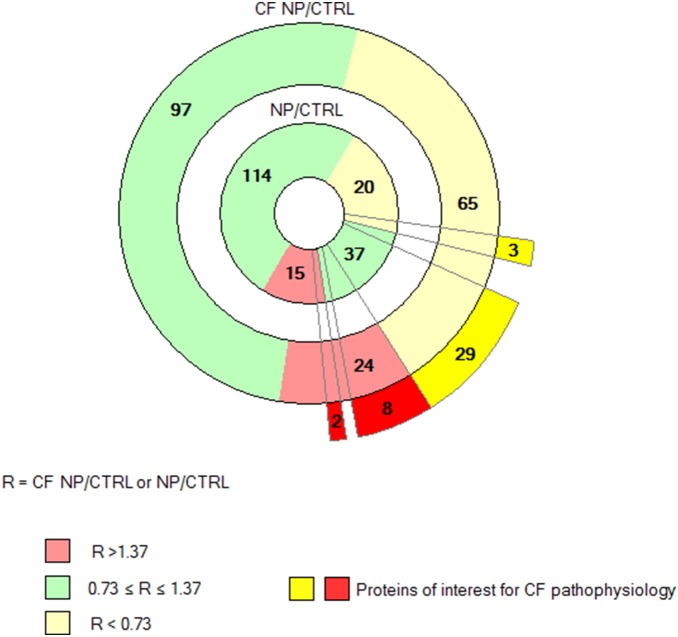
Distribution of the 186 common quantified proteins in CFNP (outer donut) and NP (inner donut) *vs* CTRL. Downregulated proteins are highlighted in yellow, upregulated proteins are highlighted in red and stable proteins are highlighted in green according to the ratio thresholds (R = CF NP/CTRL or NP/CTRL). 15 proteins are upregulated in NP compared to CTRL (inner donut) and 21 proteins are upregulated in CFNP compared to CTRL (outer donut). Among them, 11 proteins are upregulated in both CFNP and NP. 20 proteins are downregulated in NP compared to CTRL (inner donut) and 48 proteins are downregulated in CFNP compared to CTRL (outer donut). Among them, 16 proteins are downregulated in both CFNP and NP. Finally, among the proteins deregulated in common between CFNP and NP, two are more regulated in CFNP and three are more downregulated in CFNP. The 42 proteins of interest for CF pathophysiology are represented in intense red and intense yellow outside the outer donut.

**Figure 3:**
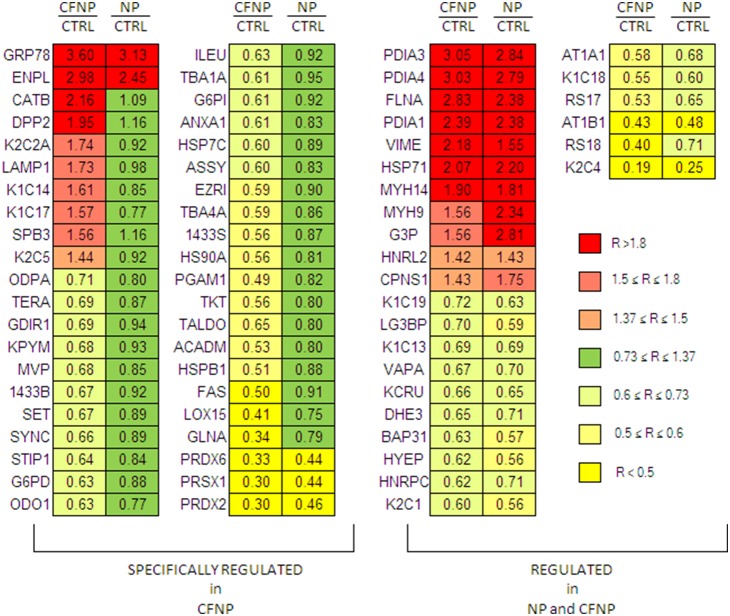
Comparison of differentially expressed proteins in CFNP compared to NP and CTRL. On the left, we report the ratios of 42 proteins significantly modulated in CFNP/CTRL *vs* NP/CTRL patients. On the right, we report proteins that are modulated in both CFNP/CTRL and NP/CTRL patients. To obtain the CFNP/CTRL ratios, relative protein expressions ratios from CF NP/NP were normalized to the average of the ratios obtained in NP *vs* CTRL (CF NP/CTRL = CFNP/NP×NP/CTRL).

**Figure 4:**
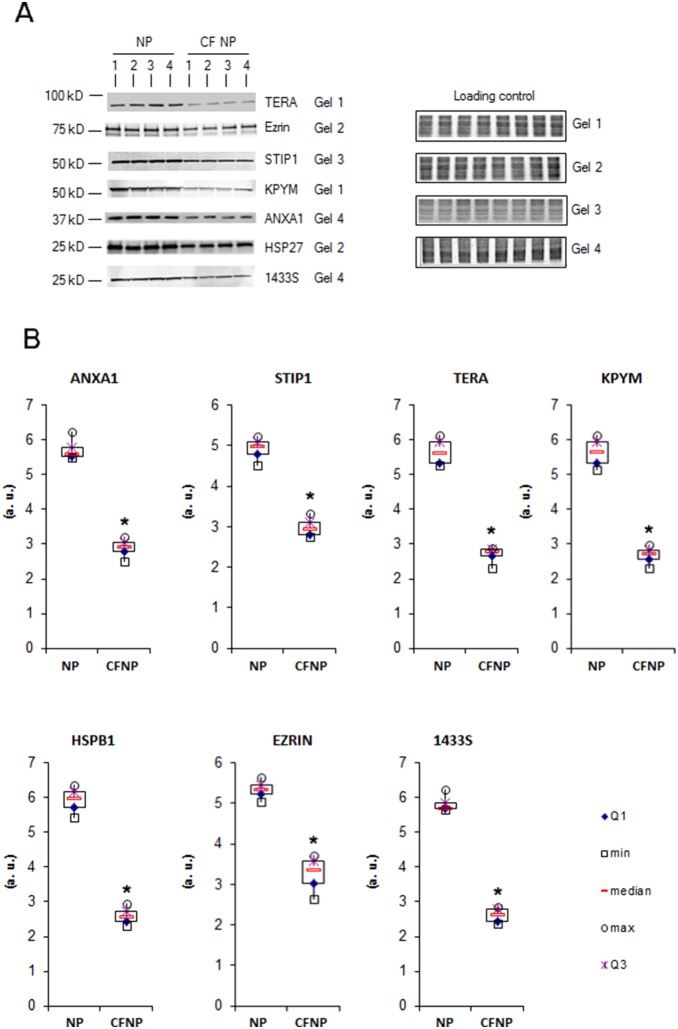
Western blot analysis of seven differentially expressed proteins. The Western blots from the 4 NP patients and the 4 CFNP patients used for proteomic experiments are shown with their respective loading controls. **A**: Each protein is analyzed in three technical replicates. For normalization, the acrylamide gels were stained with Coomassie blue (total migrated protein detection) just after electro-transfer and scanned using the Odyssey LI-COR. Using the Odyssey software, protein intensity values were normalized with their corresponding loading control intensity values. **B**: Box plots of the intensity calculated with the Odyssey software, *p<0.01, Mann and Whitney test. (a.u.: arbitrary units). We report Maximum (max), minimum (min), first quartile (Q1), third quartile (Q3).

**Figure 5:**
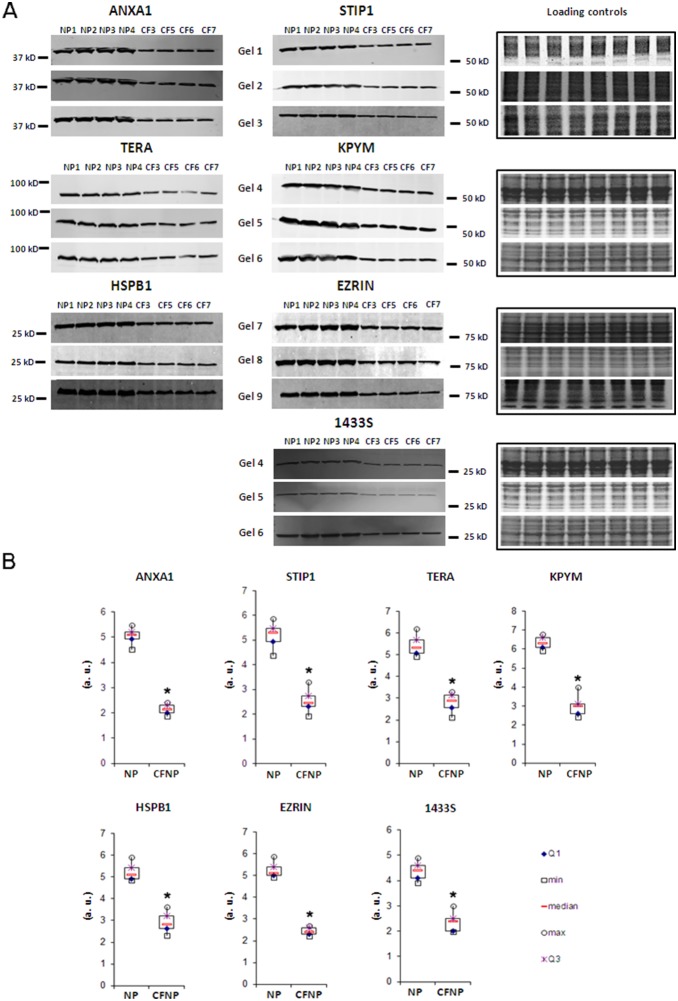
Western blot analysis of ANXA1, STIP1, TERA, KPYM, HSPB1, EZRIN and 1433S in primary cultures of human nasal epithelial cells (HNEC) from NP (n = 4) and CFNP (n = 4). **A**: Each protein was analyzed in three technical replicates. For normalization, the acrylamide gels were stained with Coomassie blue (total migrated protein detection) just after electro-transfer and scanned using the Odyssey LI-COR. Using the Odyssey software, protein intensity values were normalized with their corresponding loading control intensity values. **B**: Box plots of the intensity calculated with the Odyssey software, *p<0.01, Mann and Whitney test. (a.u.: arbitrary units). We report Maximum (max), minimum (min), first quartile (Q1), third quartile (Q3).

186 proteins were quantified across eight patients in both sets of experiments (CFNP *vs*. NP and NP *vs.* CTRL). The distribution of modulated proteins was different in CF and NP: CFNP presented a higher proportion of deregulated proteins (24 upregulated and 65 downregulated) than NP patients (15 upregulated and 20 downregulated). In order to identify the proteins that were specifically deregulated in CFNP patients, we selected proteins that are significantly modulated in CFNP patients compared to NP patients. 8 proteins were upregulated in CFNP, but stable in NP patients, 29 were downregulated in CFNP but stable in NP, 3 proteins were significantly more downregulated and 2 proteins more upregulated in CFNP than in NP (Figure 2). In addition, 11 proteins were equally upregulated and 16 proteins were equally downregulated in both CFNP and NP. The expression ratios of the modulated proteins are presented in Figure 3 (Table S1 for full details). Seven of these 42 differential proteins (TERA, EZRIN, STIP1, KPYM, ANXA1, HSP27 and 1433S) were analyzed by Western blot on patients CFNP1, 2, 3, 4 and NP1, 2, 3, 4 (Figure 4). To extend our validation we repeated the Western blot analysis, on three new additional CFNP patients (CFNP5, 6, 7). Furthermore, technical reproducibility of Western blot analysis, was also assessed by performing three technical replicates for the seven above-mentioned proteins derived from NP1, 2, 3, 4 and CFNP3, 5, 6, 7 (Figure 5A). The statistical analysis of Western blot results confirmed fully the differential expressions observed in the proteomic quantification data (Figure 5B).

### Functional distribution of the differential proteins in CFNP/CTRL

**Figure 6:**
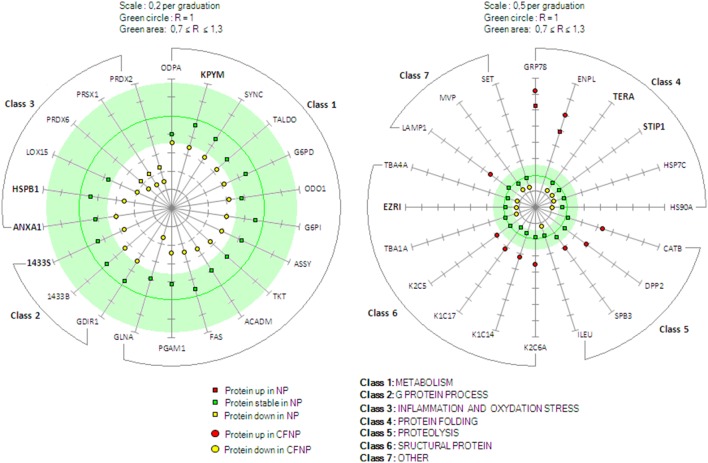
Expression pattern of the 42 differential proteins of interest in CF. The 42 differential proteins are displayed in seven different classes related to their functions (according to the AmiGO toolbox kit). Left graph: metabolism (class 1), G protein process (class 2), inflammation and oxidative stress (class 3); right graph): protein folding (class 4), proteolysis (class 5), structural protein (class 6), other (class 7). The green circle represent the absolute stable ratio value (R = 1) and the green area represent the ratio thresholds (0.73≤R≤1.37) (R = CFNP/CTRL or NP/CTRL). Scale graduations for R are 0.2 and 0.5 for left and right graph, respectively. The CFNP/CTRL ratio values of the differential proteins are represented by yellow dots (downregulated in CFNP) and red dots (upregulated in CFNP); the NP/CTRL ratio values are represented by yellow squares (downregulated in NP), red squares (upregulated in NP), and green squares (stable in NP). For the proteins in bold, the modulation was confirmed by Western blot. Protein names are from Swissprot, ratio values and p values are available in supplemental material (Table S1).

To further investigate the altered molecular mechanisms in CF epithelial cells, the differential proteins were analyzed using the AmiGO toolkit which allows highlighting the most represented classes of proteins in a given set. For the retained 42 proteins specifically modulated in CFNP, the AmiGO analysis revealed six major classes of proteins important for cell functions: metabolism, G protein process, inflammation and oxidative stress response, protein folding, proteolysis and structural proteins (Figure 6).

#### Metabolism (class 1)

**Figure 7:**
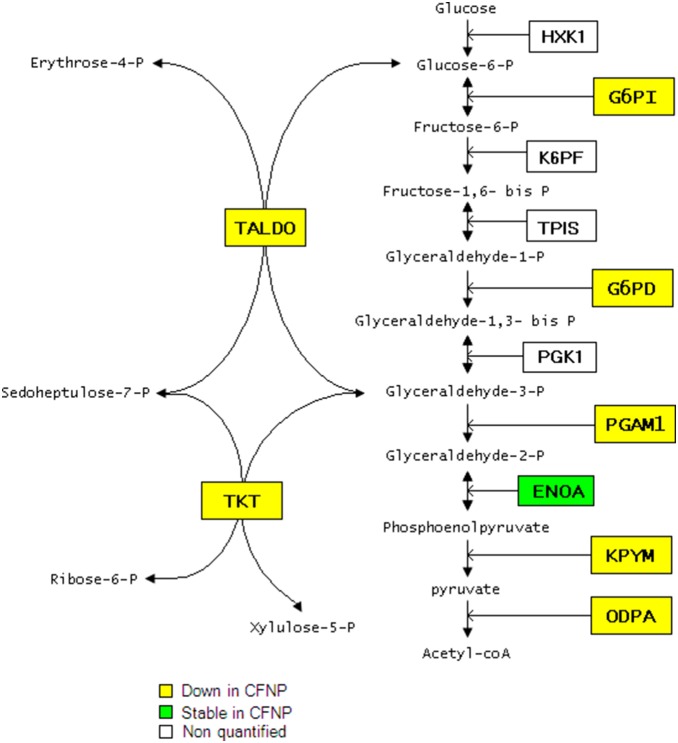
Diagram of the glycolysis/pentose pathway indicating the 7 downregulated proteins in CFNP.

Thirteen proteins implicated in metabolism were downregulated specifically in CFNP. Among them, seven proteins implicated in glycolysis related metabolism (KPYM, G6PD, ODPA, G6PI, PGAM1, TALDO and TKT) were found downregulated in CFNP and stable in NP suggesting metabolism abnormalities related to CF. Four of these proteins are directly implicated in the canonical glycolytic pathway, pyruvate kinase (KPYM), glucose phosphate isomerase (G6PI), glucose phosphate dehydrogenase 1 (G6PD) and pyruvate dehydrogenase (ODPA). Three other proteins, phosphoglycerate mutase 1 (PGAM1), transketolase (TKT) and transaldolase (TALDO), are implicated in the interconnection between glycolysis and the pentose pathway (Figure 7). In addition, four proteins implicated in amino-acid metabolism, arginosuccinate synthase (ASSY), glutamine synthetase (GLNA), asparaginyl-tRNA synthetase (SYNC), oxoglutarate dehydrogenase (ODO1), and two proteins implicated in fatty acid metabolism, medium-chain specific acyl-CoA dehydrogenase (ACADM) and fatty acid synthase, (FAS) were also significantly downregulated in CFNP compared to NP and CTRL.

#### G protein process (class 2)

Three proteins implicated in these pathways were significantly downregulated in CFNP compared to NP and CTRL, Rho GDP-dissociation inhibitor 1 (GDIR1), 14-3-3 proteins sigma and beta (1433S, 1433B), suggesting possible abnormalities in the ligand/receptor responsiveness in CF.

#### Inflammation and oxidative stress response (class 3)

Six proteins implicated in enzymatic antioxidant response and inflammation were found to be downregulated in CFNP compared to NP and CTRL. Among them, three inflammation-related proteins, arachidonate 15-lipoxygenase (LOX15), annexin A1 (ANXA1) and heat shock protein beta 1 (HSPB1) were stable in NP compared to control suggesting a CF specificity. Three proteins related to oxidative stress, the peroxiredoxins 1, 2 and 6, (PRDX1, 2 and 6), were downregulated in CFNP and NP compared to CTRL. However, these downregulations in CFNP were significantly lower compared to NP suggesting that these anomalies are present in both nasal polyposis but they are more severe in CF.

#### Protein folding (class 4)

Four protein folding-related proteins were found significantly downregulated in CFNP whilst stable in NP. Interestingly, these proteins, heat shock protein 90-alpha and cognate 71 (HS90A, HSP7C), transitional endoplasmic reticulum ATPase (TERA), stress-induced-phosphoprotein 1 (STIP1), are known to participate in proper folding and degradation of CFTR [20]–[22]. In addition, two proteins related to protein folding in endoplasmic reticulum were upregulated in CFNP with significant differences with NP, glucose-regulated protein 78 (GRP78) and endoplasmin (ENPL). These proteins are known to be upregulated in the unfolded protein response (UPR) [23].

#### Proteolysis (class 5)

Three proteins implicated in proteolysis were upregulated, serpin B3 (SPB3), cathepsin B (CATB) and dipeptidyl peptidase 2 (DPP2) and one downregulated, serpin B1 (ILEU), in CFNP and stable in NP suggesting proteolysis abnormalities related to CF.

#### Structural proteins (class 6)

Focusing on the cytoskeleton, we observed four proteins upregulated, keratins 6A, 14, 17 and 5 (K2C6A, K1C14, K1C17, K2C5) and three downregulated, ezrin (EZRI), tubulins alpha 1A and 4A (TBA1A and TBA4A) in CFNP and stable in NP compared to CTRL suggesting a cytoskeleton reshuffle in CF.

#### Other (class 7)

The HLA class II-associated protein I (SET) was downregulated in CF and is implicated in MAP kinase signaling since it regulates ERK1/2 phosphorylation [24]. We found an upregulation of the lysosome-associated membrane protein 1 (LAMP1), a well-known lysosomal membrane marker [25] suggesting an increased degradation activity in CF. Finally, the major vault protein (MVP) was downregulated in CF and is implicated in the bacterial resistance of lung epithelial cells [26].

## Discussion

In this study we found 42 proteins significantly and specifically modulated in CFNP patients. For seven of these proteins the results were validated by Western blot in the eight patients analysed by proteomics and in three additional CF patients. Our data are also supported by previously published studies by us [11], [13], [27] and by others [9], [10] showing deregulation of ANXA1 (in CFTR−/− mouse), of GRP78 for UPR activation (in both CFNP and NP), of PRDX6, PRDX2, CATB, HSPB1, PDIA3, ANXA1, VIME and EZRI (in CF cells). Importantly, we found many novel differentially expressed proteins in CF NP that could be of importance in CF pathophysiology.

**Figure 8:**
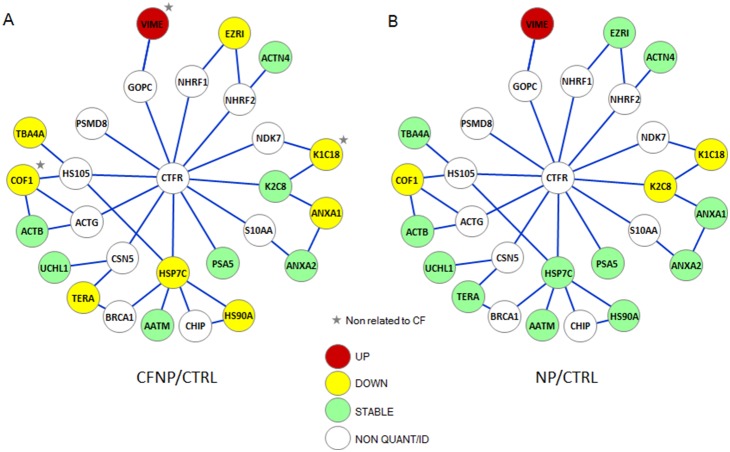
Quantified proteins present in the CFTR physical network. Proteomics quantitative analysis allowed us to reproducibly quantify 186 proteins. Of those, 16 could be mapped on a hand curated CFTR physical network based on the Biogrid interaction database (http://thebiogrid.org).This network is composed of 198 proteins selected according to experimental criteria for direct protein-protein interactions (surface plasmon resonance (SPR), pull-down and FRET experiments). To this bibliographic network, we have added proteins identified in our laboratory using SPR and DNA ligation assays. We built the CFTR protein interaction network by taking into account only proteins that directly interact with one another, at first and second degrees. In the graph, we represent the 16 proteins with a color code corresponding to their expression status in CF (**A**) and NP (**B**) compared to CTRL: upregulated (red), downregulated (yellow), stable (green). The network is completed by a minimal number of connecting proteins of the CFTR network (white) that could not be identified or quantified by MS/MS. Proteins that are up or downregulated in both CFNP and NP are marked by a grey star.

Several proteins involved in CFTR synthesis/degradation were found to be dowregulated in CF (HSP7C, HS90A, STIP1, TERA and LAMP1), supporting previous observations of alteration of folding and degradation pathways in CF [20], [21]. HSP7C, HS90A and STIP1 form a protein complex that assists correct CFTR protein folding and participates in the degradation of misfolded F508del CFTR by promoting ubiquitination [20], [21], [28]. In addition, TERA plays an essential role in endoplasmic reticulum-associated degradation [29] and is known to be involved in the ER-associated degradation of CFTR [22]. Of note, TERA, HSP7C and HS90A are present in the network of the CFTR physical interaction proteins (Figure 8). These downregulations observed in CF are surprising since F508del CFTR is mostly retained in ER for degradation and remain to be investigated. In addition, we observed an upregulation of a lysosomal membrane marker, LAMP1 [25], perhaps related to a raised turnover of the rescued F508del CFTR by the lysosomal pathway [30]. Altogether, these data may suggest a diminished folding-control/ER-degradation of F508del CFTR associated with a rise in lysosomal-mediated turnover, suggesting that F508del CFTR may be preferentially degraded by the lysosomal pathway in our model.

Several proteins involved in inflammation and oxidative stress were found to be deregulated in CF (PRDX1, 2 and 3, HSPB1, LOX15, ANXA1 and MVP), supporting previous studies [13], [31], [32]. PRDX1, 2 and 6 are implicated in enzymatic antioxidant defenses and we have previously shown a downregulation of PRDX6 under oxidative challenge in CFTR−/− mice lungs [13]. In another study, PRDX6 has also been found to be downregulated in HNEC of CF patients [10]. Another protein linked to oxidative stress, HSPB1 is able to raise intracellular glutathione levels [33] and activate ROS detoxification enzymes such as the peroxiredoxins mentioned above [34]. The decreased expression of these proteins in CFNP may result in lower protection against oxidative stress suggesting a susceptibility to oxidative damage in CF. LOX15 produces 15-hydroxyeicosatetraenoic acid (15-HPETE), an inhibitor of inflammatory mediators by promoting TNF mRNA decay [35]. ANXA1 is also known to have anti-inflammatory properties [36]. The downregulation of these two proteins participates in inflammation in CF by deregulating the eicosanoid-related pathway. Interestingly, MVP is known to participate in the resistance of airway cells to bacteria and may participate in the well-known lower resistance of CF patients to Pseudomonas aeruginosa infection [26].

Eight proteins belonging to the glucose and fatty acid metabolic pathways were found to be deregulated in CF (KPYM, G6PI, G6PD, ODPA, PGAM1, TALDO, TKT and ACADM). KPYM, G6PI, G6PD, ODPA, PGAM1, TALDO and TKT are directly implicated in the glycolysis/pentose pathway leading to NADPH and acyl-CoA production (Figure 7). Interestingly, a recent metabolomic study showed a diminished amount of glycolysis-related metabolites in CF cells (*i.e.* glucose, glucose-6-phosphate, fructose-6-phosphate) and concluded that glycolysis could be suppressed in CF [12]. However, a recent study showed upregulations of KPYM in IB3-1 cells compared to C38 cells, concluding that glycolysis could be increased in CF [8]. This discrepancy could be related to the differences between primary cells and transfected cellular lineages. ACADM is the medium chain of acyl-CoA dehydrogenase that is a key enzyme of fatty acid beta-oxidation leading to acyl-CoA production. The protein expression and activity of all these metabolic enzymes have never been directly investigated in CF. Altogether, our data show that the two major pathways leading to the production of NADPH and acetyl-CoA seem to be altered in CF and could further aggravate oxidative stress conditions.

Deregulation of proteases (SPB3 and CATB) and of a protease inhibitor (ILEU) may participate in lung fibrosis frequently observed in CF. SPB3, ILEU and CATB are important inflammation modulators. SPB3 and CATB (upregulated in this study) have been shown to promote lung, intestinal and liver fibrosis, respectively, in mouse models [37], [38]. Furthermore, ILEU (downregulated in this study) is a leukocyte elastase inhibitor that prevents lung fibrosis in mice [39]. These anomalies, detected by us in upper airway epithelial cells, may represent at the cellular level the molecular anomalies that could lead to lung fibrosis. In addition, the upregulation of cytoplasmic structural proteins in CF (K2C5, K1C14, K2C6A and K1C17) and the downregulation of TBA1A, TBA4A and EZRI, known to interact with CFTR by anchoring it to the tubulin cytoskeleton [40], [41], suggest a possible intracellular remodeling of airway epithelial cells that could be related to trafficking and/or stabilization processes. All together, these results suggest a possible remodeling at the intra and extra cellular levels.

G protein related pathways were found to be altered in CF, suggesting numerous dysfunctions in CF cells. Three proteins related to G protein signaling (GDIR1, 1433B and 1433S) and one protein related to MAP kinase signaling (SET) (all downregulated) are multifunctional proteins that regulate most of the major cellular functions including proliferation, apoptosis, inflammatory response, epithelial cell polarization and secretory processes [42]. Those downregulations could be related to some or many alterations described above.

Finally, based on the Biogrid interaction database (http://thebiogrid.org), we built the CFTR protein interaction network by taking into account only proteins that directly interact with one another, at first and second degrees. This network is composed of 198 proteins selected according to experimental criteria for direct protein-protein interactions (surface plasmon resonance (SPR), pull-down and FRET experiments). To this bibliographic network we have added proteins identified in our laboratory using SPR and DNA ligation assays [43]. It is of interest to note that 16 of the 186 quantified proteins in the present study could be mapped on our handcurated CFTR physical network (Figure 8). Remarkably, 9 proteins out of these 16 were deregulated in CF patients (8 down and 1 up), while only 4 of the 16 proteins were downregulated (3) or upregulated (1) in NP patients (Figure 8). Those differentially expressed proteins are cytoskeleton or chaperon proteins that are all implicated in the folding, stability or degradation of CFTR. We suggest that some of the deregulated proteins in CF patients may be related to the perturbation of the CFTR/F508delCFTR physical network.

Overall, our proteomic study provides a reproducible set of differentially expressed proteins in airway epithelial cells from CF patients. While some of our findings correlate with previous studies, we have identified many novel differentially expressed proteins that have not been previously studied in CF pathophysiology.

## Supporting Information

Table S1 **List of quantified proteins:** Ratio values, Mann-Whitney test (p-value<0.05) and Student test with Aspin-Welch correction (p-value<0.05).
(PDF)

Table S2 **Antibodies used in this study:** Target, stock concentration, dilution used, manufacturer, reference, host and clonality.
(XLSX)
